# COGNITIVE TRAINING ATTENUATES AGE-RELATED DECLINE IN PHYSICAL FUNCTION ACROSS 10 YEARS

**DOI:** 10.1093/geroni/igz038.785

**Published:** 2019-11-08

**Authors:** Briana N Sprague, Christine B Phillips, Lesley A Ross

**Affiliations:** 1 The Pennsylvania State University, University Park, Pennsylvania, United States; 2 Arizona State University, Arizona State University, United States

## Abstract

Poor physical function is associated with negative health and cognitive outcomes. Although nine studies demonstrate that cognitive training reduces age-related declines in physical function, only one has examined effects beyond immediate posttest changes. The current study assessed the impact of three cognitive training programs on physical function measures across 10 years. Using data from the Advanced Cognitive Training for Independent and Vital Elderly (ACTIVE) trial, older adults randomized to a no-contact control condition (n = 698) were compared to those randomized to processing speed (n = 702), memory (n = 703), or reasoning (n = 694) training. Intention-to-treat and treatment-received analyses were conducted for grip strength, Digit Symbol Copy, and Turn 360. There were no significant effects of being assigned to processing speed, memory, or reasoning training to any physical function outcome (p > .05). Treatment-received models indicated that processing speed training attenuated age-related declines in Digit Symbol Copy (b = -.005, p < .01) and Turn 360 (b = -.011, p < .001), memory training attenuated age-related declines in Digit Symbol Copy (b = -.009, p < .001) and Turn 360 (b = -.011, p < .001), and reasoning training attenuated age-related declines in Digit Symbol Copy (b = -.012, p < .001) and Turn 360 (b = -.012, p < .001). There was no significant transfer to grip strength. This is the first study to demonstrate beneficial effects of cognitive training to some physical functions across 10 years. Future work should examine moderators and mediators of transfer effects.

